# Health Tract, No. 197

**Published:** 1865

**Authors:** 


### HEALTH TRACT, No. 197.

### THE FAITHLESS PHYSICIAN.

On Saturday the eighth day of October, 1864, a rich man was taken to his grave from the city of New York, where, by a life of industry, temperance, economy, and the exercise of a sound judgment, he had acquired a large estate; not suddenly, but in the progress of thirty years. From some obscure cause, he became a great sufferer from general neuralgia. A physician was called, but in the progress of weeks, did not afford the invalid that speedy and decided relief, which he and his friends so much desired. He wanted more potential remedies, and was not willing to submit to those restraints to system and rule in living, which the experience of years had taught his medical attendant were of the highest value ultimately. Another physician was called in, who advised a different course of procedure. He said the man needed strength and flesh and vigor; that these could only be secured by a generous diet and a liberal allowance of stimulants at every meal. Roast beef and the best brandy were regularly provided: the neuralgia was steadily abating and a speedy restoration was looked for. Still, days passed on, and gentlemen calling at his counting house were informed that, The Head of the firm was "out of town." This answered very well for a while, but a gentleman who had large business engagements with the invalid, found it absolutely necessary to save a ruinous loss, to have a personal interview; after a variety of obstacles were overcome the rich man was found at his own home in the city, the victim of the most fearful form of Delirium Tremens. Brandy had given relief; but it was soon found that unless it was administered more frequently, and in larger quantities the pains would return, hence there was an unbridled indulgence in the remedy, the result being, that at the end of a few weeks, it was found necessary to keep the patient always under the influence of liquor, that is, always drunk; at length however, the outraged stomach refused to retain the fiery stimulant a single moment, while for want of it a raving delirium sat in, ending in unconsciousness and death, before the business could be consummated by his intelligent signature. Here was a man who, temperate all his life, had died a drunkard's death as the result of an unwise medical prescription, the fashionable and almost universal prescription of a certain class of so called "Doctors," of "Bourbon Whiskey;" and there are thousands of deluded persons of every age and sex, especially in New England, on the same road to ruin. A few years ago, cod liver oil was given for everything; now it is Brandy. Shame on the medical colleges of the land, which send out annually so many incompetent and unprincipled graduates.

